# STAT6/VDR Axis Mitigates Lung Inflammatory Injury by Promoting Nrf2 Signaling Pathway

**DOI:** 10.1155/2022/2485250

**Published:** 2022-01-10

**Authors:** Youjing Yang, Qianmin Li, Shuhui Wei, Kaimiao Chu, Lian Xue, Jie Liu, Yu Ma, Shasha Tao

**Affiliations:** ^1^School of Public Health, Medical College of Soochow University, 199 Ren'ai Road, Suzhou 215123, China; ^2^The Fifth People's Hospital of Suzhou, The Affiliated Infectious Hospital of Soochow University, 10 Guangqian Road, Xiangcheng District, Suzhou 215131, China; ^3^Chongqing University Central Hospital & Chongqing Emergency Medical Center, No. 1 Jiankang Road, Yuzhong District, Chongqing 400014, China

## Abstract

Lung inflammatory injury is a global public health concern. It is characterized by infiltration of diverse inflammatory cells and thickening of pulmonary septum along with oxidative stress to airway epithelial cells. STAT6 is a nuclear transcription factor that plays a crucial role in orchestrating the immune response, but its function in tissue inflammatory injury has not been comprehensively studied. Here, we demonstrated that STAT6 activation can protect against particle-induced lung inflammatory injury by resisting oxidative stress. Specifically, genetic ablation of STAT6 was observed to worsen particle-induced lung injury mainly by disrupting the lungs' antioxidant capacity, as reflected by the downregulation of the Nrf2 signaling pathway, an increase in malondialdehyde levels, and a decrease in glutathione levels. Vitamin D receptor (VDR) has been previously proved to positively regulate Nrf2 signals. In this study, silencing VDR expression in human bronchial epithelial BEAS-2B cells consistently suppressed autophagy-mediated activation of the Nrf2 signaling pathway, thereby aggravating particle-induced cell damage. Mechanically, STAT6 activation promoted the nuclear translocation of VDR, which increased the transcription of autophagy-related genes and induced Nrf2 signals, and silencing VDR abolished these effects. Our research provides important insights into the role of STAT6 in oxidative damage and reveals its potential underlying mechanism. This information not only deepens the appreciation of STAT6 but also opens new avenues for the discovery of therapies for inflammatory respiratory system disorders.

## 1. Introduction

Lung inflammatory injury is a common pathological feature of various pulmonary diseases, and it poses a huge economic and social burden worldwide [1]. Its clinical features include infiltration of diverse inflammatory cells and thickening of pulmonary septum along with oxidative stress to airway epithelial cells [2]. This disease has diverse pathogenic factors, such as environmental pollutants (particulate matter (<2.5microns in aerodynamic diameter)), drug side effects, and viral or fungal infection [3–5]. Uncontrolled inflammation is reportedly one of the determinants that induce thoracic complication in patients with coronavirus disease 2019 [6]. Recently, the incidence of lung inflammatory injury has sharply increased, but effective therapeutic strategies for this disease are limited. Therefore, the mechanism and pathogenesis of pulmonary inflammatory disorder must be understood, and novel and effective means of prevention/intervention should be developed.

The members of the signal transducer and activator of transcription (STAT) family of proteins, which consists of STAT1, STAT2, STAT3, STAT4, STAT5a, STAT5b, and STAT6, are vital regulators in organisms [7]. Among these members, STAT6 reportedly strongly regulates immune responses, which activate and promote responsive gene transcription through Janus kinase 3-mediated phosphorylation by Th2 cytokines, such as IL4 and IL13 [8–10]. Extensive studies have highlighted that STAT6 orchestrates major immunoregulation mechanisms in various inflammatory diseases, and one of its cornerstones is its effects on T cells [11]. Moreover, STAT6 can polarize macrophages toward anti-inflammatory M2 phenotype, thereby mediating the resolution of inflammation and repair [12–14]. Inflammation-induced tissue oxidative stress is a major factor that causes cell damage [15]. Timely removal of reactive oxygen species (ROS) can attenuate tissue injury [16, 17]. However, the role of STAT6 in inflammation-induced tissue oxidative stress and the issue of whether it can directly prevent against oxidative stress-related cell apoptosis are unclear.

The transcription factor NFE2-related factor 2 (Nrf2) is an emerging therapeutic target for several diseases. In general, Nrf2 can regulate numerous antioxidative, anti-inflammatory, and prosurvival genes to neutralize free radicals and accelerate the removal of environmental toxins [18, 19]. Inflammatory injury is widely known to be always associated with ROS generation, leading to increased levels of oxidative DNA damage and cell apoptosis [20–22]. Thus, Nrf2 activation may be a potential strategy for treatment of lung injury. Nrf2 activation involves both canonical and noncanonical mechanisms. The former mainly entails modifying Keap1 cysteine residues, whereas the latter concerns the activation of Nrf2 in an autophagy-dependent manner involving p62. Specifically, p62 (the adaptor of autophagy) competitively binds with Keap1, which causes Keap1 degradation in autophagolysosomes, thereby activating Nrf2 [23, 24]. Liu et al. [25] found that spermidine can induce the Nrf2 signaling pathway through MAP1S, which accelerates p62-dependent Keap1 degradation via the autophagy pathway. Similarly, Bae et al. [26] reported that sestrins activate Nrf2 by promoting p62-dependent autophagic degradation of Keap1 and prevent oxidative liver damage. Although numerous studies have determined that Nrf2 can be regulated by many proteins in numerous biological processes, such as PML-RAR*α*, the question of whether it is regulated by STAT6 has not been studied thus far [27, 28].

In this study, we demonstrated that STAT6 can positively regulate the Nrf2 signaling pathway both *in vivo* and *in vitro*. Furthermore, we revealed that STAT6 regulates autophagy. Specifically, we observed that STAT6 activation promotes the translocation of VDR to the nucleus, which increases the transcription of autophagy-related downstream genes, subsequently inducing Nrf2 signaling. Our research provides important insights into the role of STAT6 in oxidative damage and reveals its regulative mechanism in Nrf2 signaling. This information not only deepens the appreciation of STAT6 but also opens new avenues for the discovery of therapies for inflammatory airway disorders.

## 2. Materials and Methods

### 2.1. Antibodies and Cell Culture

The primary antibodies purchased from Santa Cruz Biotechnology are specified as follows: anti-STAT6: sc-374021, anti-p-STAT6: sc-136019, anti-Nrf2: sc-13032, anti-NQO1: sc-32793, anti-VDR: sc-13133, anti-CYP24A: sc-365700, anti-Histone H3: sc-517576, anti-Tublin: sc-8035, anti-ATG7: sc-376212, anti-Beclin1: sc-48381, and anti-GAPDH: sc-32233. Antibody against LC3B was purchased from novusbio (NB100-2220). Secondary antibodies conjugated with horseradish peroxidase (HRP) were from Immunoway (Plano, TX: RS0001 (Mouse) and RS0002 (Rabbit)). Human bronchial epithelial cell BEAS-2B and human acute monocytic leukemia cell line (THP-1) were purchased from ATCC (Manassas, VA). BEAS-2B cells were cultured in BEBM with additives from Lonza/Clonetics Corporation (CC-3170), and THP-1 cells were cultured in RPMI 1640 medium supplemented with 10% FBS (Hyclone, Logan, UT) and 0.1% gentamycin (Invitrogen, Carlsbad, CA). For the differentiation of THP-1 cells, 5 ng/ml phorbol-12-myristate-13-acetate (PMA) obtained from Sigma was used. The cells were maintained at 37°C in a humidified incubator containing 5% CO_2_.

### 2.2. Particle Preparation

The particles of Quartz DQ 12 were obtained from Doerentrup Quarz GmbH (Germany), and the content of the free SiO_2_ was more than 99%. The detail particle diameter is as follows: 90% less than 2.3 *μ*m, 50% less than 1.1 *μ*m, and 10% less than 0.6 *μ*m. The particles were grinded in saline for 3 h. After boiling in 1 N HCl, the particles were resuspended with sterile saline. Finally, sonication was employed to particles for 10 min before use.

### 2.3. Animal Work

Six to 8-week-old C57BL/6 mice were purchased from SLAC Laboratory Animal Co., Ltd., Stat6^flox/flox^ mice, and knockin Sftpc^Cre^ mice were purchased from Cyagen Biosciences. To generate lung epithelium-specific Stat6 deficient mice, Stat6^flox/flox^ mice were crossed with Sftpc^Cre^ mice. Then, tamoxifen was injected accordingly to induce the activity of Cre [29]. Eight-week-old gender matched wildtype mice Stat6^flox/flox^ (design as Stat6^+/+^) and knockout mice Stat6^flox/flox^Sftpc^Cre^ (design as Stat6^−/−^) from the same litter were selected randomly to indicated groups based on genotypes. Genotyping analyses were performed by PCR with genomic DNA isolated from mouse tails. All mice, between 8 and 12 weeks old, were obtained in 12 h/12 h light-dark cycle, pathogen-free condition with water and food ad *libitum*. Both wildtype and Stat6^−/−^ mice were randomly separated into two groups (*n* = 6 per group): control (intratracheally instilled with 50 *μ*l sterile saline, defined as Ctrl) and silica particles (intratracheally instilled with the particles suspension, 3 mg silica in 50 *μ*l sterile saline, defined as particle). Mice were monitored and sacrificed at day 7 following particle instillation. The study protocols followed the Guide for the Care and Use of Laboratory Animals were approved by Soochow University Institutional Animal Care and Use Committee.

### 2.4. H&E and Immunohistochemistry (IHC) Staining

Paraffin-embedded lung tissues were cut, baked, and deparaffinized. Then, the lung tissue sections (4 *μ*m) were subjected to hematoxylin and eosin (H&E) staining. Also, prepared lung tissues were sectioned and analyzed by immunohistochemistry using specific antibodies. Briefly, after antigen retrieval, the slides were incubated with primary antibodies at 4°C overnight. Secondary antibodies were added for 20 min at 37°C. Images were acquired using a fluorescence microscope. The staining of 8-oxo-dG was performed as described previously [30].

### 2.5. Bronchial Alveolar Lavage Fluid (BALF) Analysis

Mice were euthanized, and bronchoalveolar lavage fluid (BALF) was obtained by lavaging the lung with 1 mL cold PBS through the tracheal cannula. The BALF was centrifuged, and the supernatant was collected. The total protein concentration in BALF was determined by the BCA protein assay kit based on the manufacturer's instructions (Beyotime, Shanghai, China).

### 2.6. TUNEL Assay

TUNEL assay was performed using a One-Step TUNEL Assay Kit (Beyotime, C1086). Briefly, following 4% paraformaldehyde fixation for 30 min at room temperature, frozen sections of lung tissue were stained with 50 *μ*l TUNEL staining solution at 37°C for 60 min. Then, photographs were obtained using a fluorescence microscope.

### 2.7. Quantitative Real-Time PCR Analysis

Total RNA was extracted from lung tissues and BEAS-2B cells using TRIzol reagent and reverse transcribed into cDNA using HiFiScript cDNA synthesis kit according to the manufacturer's instructions. Equivalent amounts of each cDNA sample were added to a SYBR Green Master Mix Kit with 96-well PCR plates and 8-strip tubes (#403022, Nest, China). The primer pairs used in our study are described in Table 1.

### 2.8. Malondialdehyde (MDA) Measurement

Malondialdehyde content was measured using Lipid Peroxidation (MDA) Assay Kit (Beyotime, China, Shanghai) according to the manufacturers' instructions. In details, lysis buffer was added to lung tissue, and after homogenization and centrifugation, supernatants were blended with thiobarbituric acid (TBA) detection solution and heated at 100°C for 15 min. The absorbance at 532 nm was measured, and the MDA content was calculated according to the standard curve.

### 2.9. Glutathione (GSH) Assay

The levels of GSH were measured using the Reduced glutathione (GSH) assay Kit (A006-2-1, Nanjing Jiancheng) in accordance with the manufacturer's instructions. And the results were recorded using a microplate reader (Biotek, Seattle, United States) at 405 nm.

### 2.10. Cell Viability Assay

MTT (3-(4,5-dimethylthiazol-2-yl)-2,5-diphenyltetrazolium bromide) assay was performed to detect cell viability. Briefly, 20 *μ*l MTT (2 mg/ml) was administrated followed by incubation at 37°C for 2 hours. About 100 *μ*l of isopropanol/HCl was added into each well after MTT incubation. Then, the absorbance at 570 nm was measured using a Synergy 2 Multimode Microplate Reader (Biotek, Seattle, United States).

### 2.11. Immunoblotting, Immunofluorescence, Immunoprecipitation Assay

For immunoblotting experiments, protein was extracted from lung tissues and cells using lysis buffer containing 50 mM Tris-HCl (pH 6.8), 2% sodium dodecyl sulfate (SDS), 10% glycerol, 100 mM dithiothreitol (DTT), and 0.1% bromophenol blue. After sonication, equal amount of protein sample was resolved by sodium dodecyl sulfate-polyacrylamide gel electrophoresis (PAGE) and transferred onto a nitrocellulose membrane. And then, the membranes were incubated with indicated primary antibodies and appropriate secondary antibodies. The relative immunoblot bands are compared using the prestained protein marker (Vazyme Biotech Co., Ltd., MP102-01).

For immunofluorescence, BEAS-2B cells were grown on round glass cover slips (Fisher Scientific). With indicated treatments, cells were fixed on cover slips using chilled methanol for 15 min. After washing with PBS three times, cells were incubated with the primary antibodies and the respective fluorescent secondary antibodies for 50 min each. Cells were the mounted to glass slides and imaged. All images were observed under a fluorescence microscope.

For immunoprecipitation, cells were lysed on ice with RIPA buffer (Thermo). After centrifugation, supernatant was collected and incubated with the indicated antibodies supplemented with agarose beads (Invitrogen) overnight at 4°C with constant rotation. Immunocomplexes were washed three times using RIPA buffer with protease inhibitor and visualized by western blot with the indicated antibodies.

### 2.12. Nuclear and Cytoplasmic Extraction

The nuclear and cytoplasmic proteins were isolated from human BEAS-2B cell using Nuclear/Cytosolic Extraction Kit (CWBiotech, China, CW0199S) in accordance with the manufacturer's instructions. Briefly, cells were treated with Nc-Buffer A, and Nc-Buffer B was subsequently added. After centrifugation, the cytoplasmic protein was collected. Nuclear protein was also obtained after using Nc-Buffer C. All the Nc-Buffer were added 1% protease inhibitor before use. Both nuclear and cytoplasmic proteins were subjected to western blot analysis.

### 2.13. Statistics

Results are presented as fold changes to control groups. Data are shown as mean ± standard (SD) of three independent experiments performed in triplicates. Comparison in two groups was analyzed using two-tailed Student's *t*-test, and multiple comparison analysis was calculated by one-way ANOVA with *Bonferroni's* correction. A value of *P* < 0.05 was considered statistically significant.

## 3. Results

### 3.1. Genetic Ablation of STAT6 Sensitizes Lung to Particle-Induced Oxidative Damage in Mice

**Figure 1:**
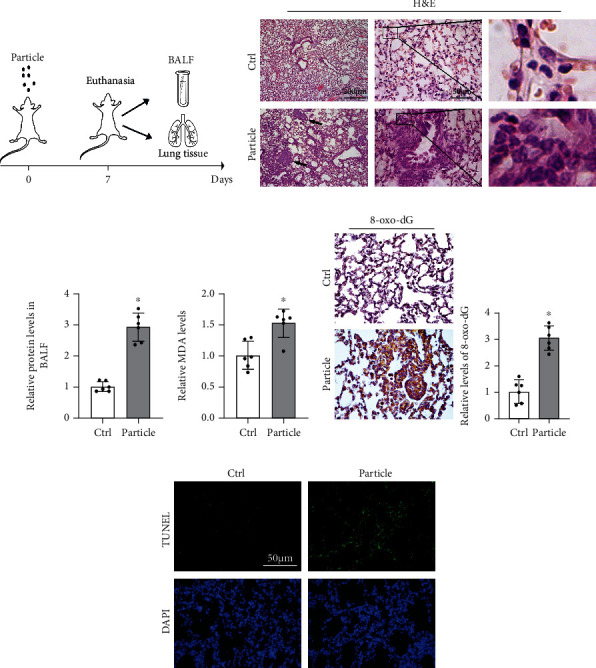
Particle caused severe oxidative injury in lung tissue. (a) Pattern of mouse model procedure. Mice received particles intratracheally at day 0 and sacrificed at 7th day. BALF and lung tissue were harvested for next analysis. (b) Representative H&E-stained lung sections (*n* = 6, black arrows indicated pulmonary nodule, and infiltration of inflammatory cells was magnified at right panel). (c) The protein content of BALF was measured. Results were shown as means ± SD (*n* = 6, ^∗^*P* < 0.05, Ctrl vs. particle). (d) Relative values of MDA in lung tissue from each group were measured. Results were expressed as means ± SD (*n* = 6, ^∗^*P* < 0.05, Ctrl vs. particle). (e) IHC staining of 8-oxo-dG in the lung tissue from the indicated group was performed and quantified. Representative images from each group were shown. Results were expressed as means ± SD (*n* = 6, ^∗^*P* < 0.05, Ctrl vs. particle). (f) Representative images of TUNEL assay staining in lung tissue from each group were shown.

To evaluate the effect of STAT6 on lung inflammation, a murine model was established with fine particle intratracheal instillation. After 7-day monitoring, mice were sacrificed, and lung tissue and bronchial alveolar lavage fluid (BALF) were collected (Figure 1(a)). H&E staining showed that lung tissue exhibited obvious pathological change after particle inhalation, manifesting as thicken alveolar septum, pulmonary nodule, and infiltration of inflammatory cells (Figure 1(b)). Also, the BALF in the particle group was detected higher level of protein compared to the control group (Figure 1(c)). Next, we examined the oxidative damage in lung tissue. MDA, the biomarker of oxidative stress, was upregulated in the particle treatment group (Figure 1(d)). Increased level of 8-oxo-dG in the particle group also indicated the DNA damage (Figure 1(e)). As exception, particle exposure increased the level of apoptosis, measured by TUNEL assay (Figure 1(f)). All these data suggested that particle instillation induced an obvious airway inflammatory injury with conspicuous oxidative stress.

**Figure 2:**
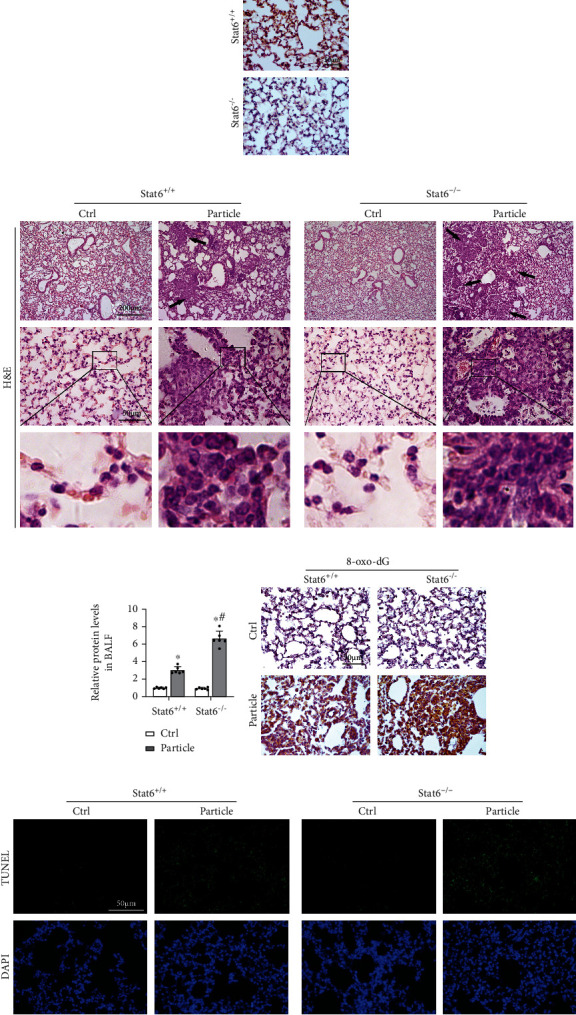
STAT6 deficiency exacerbated particle caused lung oxidative injury. (a) Representative photographs of lung tissue STAT6 staining from Stat6^+/+^ and Stat6^−/−^ mice. (b) Representative H&E-stained lung sections (black arrows indicated pulmonary nodule, and infiltration of inflammatory cells was magnified at the below). (c) The protein content of BALF from the indicated group was measured. Results were expressed as means ± SD (*n* = 6, ^∗^*P* < 0.05, Ctrl vs. particle; ^#^*P* < 0.05, Stat6^+/+^ vs. Stat6^−/−^). (d) IHC staining of 8-oxo-dG in lung tissue sections from the indicated groups was performed. (e) TUNEL staining of lung tissue. Representative images from each group were shown.

Next, lung epithelium-specific knockout mice (design as Stat6^−/−^) were further subjected to evaluate the role of STAT6 in response to particle-induced oxidative damage (Figure 2(a)). As shown in Figure 2(b), Stat6^−/−^ mice got more serious pathological damage. Also, the protein levels of BALF were higher in Stat6^−/−^ mice after exposing to particle (Figure 2(c)). Not surprisingly, Stat6^−/−^ mice showed higher levels of 8-oxo-dG than Stat6^+/+^ mice (Figure 2(d)). Likewise, Stat6^−/−^ mice exhibited more increase of apoptosis than wildtype counterparts after particle inhalation (Figure 2(e)). Overall, these murine experiments indicated that STAT6 deficiency contributed particle-induced lung oxidative injury.

### 3.2. STAT6 Deficiency Inhibits the Antioxidant Capacity through Downregulating Nrf2 Signaling Pathway

**Figure 3:**
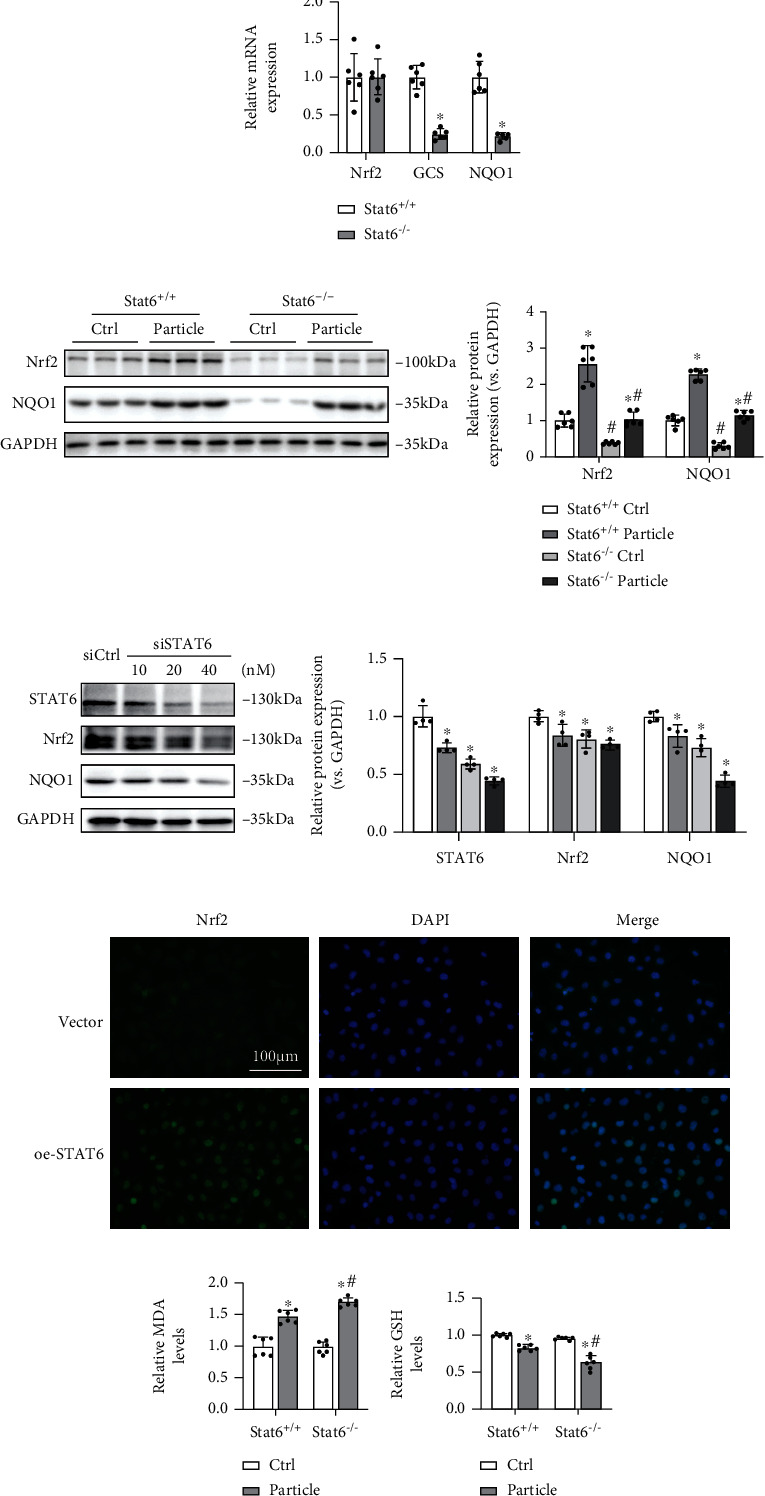
Deficiency of STAT6 impaired the antioxidant capacity of lung tissues by downregulating Nrf2 signaling. (a) The mRNA expressions of Nrf2, GCS, and NQO1 in lung tissues were detected by qRT-PCR. Results were expressed as means ± SD (*n* = 6, ^∗^*P* < 0.05, Stat6^+/+^ vs. Stat6^−/−^). (b) The protein expression levels of Nrf2 and NQO1 in lung tissues were determined by western blot analysis in the indicated group. Results were presented as means ± SD (*n* = 6, ^∗^*P* < 0.05, Ctrl vs. particle; ^#^*P* < 0.05, Stat6^+/+^ vs. Stat6^−/−^). (c) The protein expressions of STAT6, Nrf2, and NQO1 in BEAS-2B cells treated with siCtrl or indicated doses of siSTAT6 were measured using western blot analysis. Results were expressed as means ± SD (*n* = 4; ^∗^*P* < 0.05, Ctrl vs. treatment groups). (d) Immunofluorescence staining of Nrf2 in the cells with transfection of STAT6 plasmid or vector. Nuclei were stained with DAPI (blue). (e) Relative MDA and (f) GSH levels in lung tissue were measured. Results were expressed as means ± SD (*n* = 6, ^∗^*P* < 0.05, Ctrl vs. particle; ^#^*P* < 0.05, Stat6^+/+^ vs. Stat6^−/−^).

Since Stat6^−/−^ mice exhibited more sensitivity to particle-induced lung damage, Nrf2 signaling as the typical antioxidative stress factor was measured at both transcriptional and translational levels. Relative qRT-PCR results showed that the expression of Nrf2 was unchanged, while its downstream genes such as NQO1 and GCS were suppressed in Stat6^−/−^ mice (Figure 3(a)). Relative immunoblot analysis results suggested that Nrf2 signaling pathway was consistently downregulated in Stat6^−/−^ mice (Figure 3(b)). In order to further confirm these phenomena, *in vitro* experiments were carried out. After transfection with STAT6 small interfering RNAs (siRNAs), BEAS-2B cells were harvested for immunoblot analysis. The results showed that Nrf2 signaling pathway was downregulated with STAT6 suppression (Figure 3(c)). Conversely, overexpression STAT6 increased the nuclear localization of Nrf2, which indicated that STAT6 activated Nrf2 signaling pathway at the posttranscriptional levels (Figure 3(d)). These results demonstrated that genetic ablation of STAT6 can slash the antioxidant capacity of mice, making them sensitize to particle-induced lung injury, exhibiting higher levels of MDA and lower level of GSH (Figures 3(e) and 3(f)).

### 3.3. STAT6 Positively Regulates Autophagy Based on VDR

**Figure 4:**
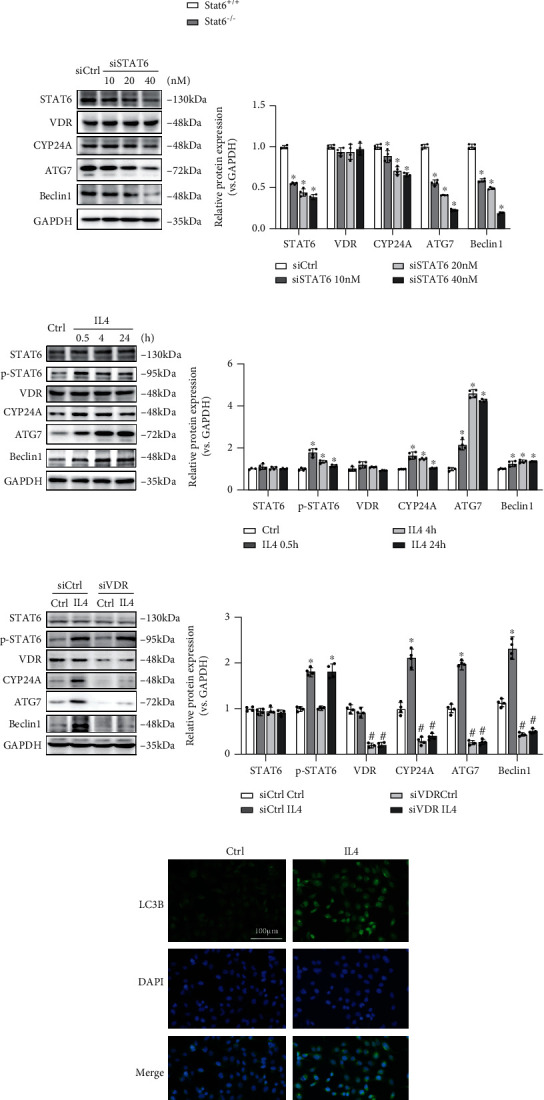
STAT6 positively regulates autophagy through VDR. (a) The mRNA levels of STAT6, Arg1, VDR, CYP24A, ATG7, Beclin1, and LC3B in lung tissue were measured by qRT-PCR assay. Results were expressed as means ± SD (*n* = 6, ^∗^*P* < 0.05, Stat6^+/+^ vs. Stat6^−/−^). (b) The protein expressions of STAT6, VDR, CYP24A, ATG7, and Beclin1 in BEAS-2B cells following different doses of siSTAT6 were measured using western blot analysis (*n* = 4; ^∗^*P* < 0.05, Ctrl vs. treatment groups). (c) The protein expressions of STAT6, p-STAT6, VDR, CYP24A, ATG7, and Beclin1 in BEAS-2B cells following different doses of IL4 were measured using western blot analysis (*n* = 4; ^∗^*P* < 0.05, Ctrl vs. treatment groups). (d) Cells were transfected with siVDR for 24 h and followed by IL4 (20 ng/ml, 24 h) treatment. The protein expressions of STAT6, p-STAT6, VDR, CYP24A, ATG7, and Beclin1 were detected by western blot analysis (*n* = 4; ^∗^*P* < 0.05, Ctrl vs. IL4; ^#^*P* < 0.05, siCtrl vs. siVDR). (e) Immunofluorescence staining of LC3B in the cells with IL4 treatment. Nuclei were stained with DAPI (blue).

On the basis of established evidence that autophagy is one of the determining factors during the activation of Nrf2, we hypothesized that STAT6 activation may positively regulate autophagy, thereby activating Nrf2 signals. Besides, previous studies have also reported that VDR can directly regulate numbers of autophagy key genes' transcription. Thus, we measured related genes' mRNA levels in mice lung tissue. The results showed that the expression of VDR and autophagy-related genes (such as ATG7, Beclin1, and LC3B) was lower in Stat6^−/−^ mice (Figure 4(a)). Meanwhile, BEAS-2B cells were transfected with STAT6 siRNA or treated with IL4 (STAT6 inducer) to suppress or induce STAT6 signaling pathway, respectively. The results showed that CYP24A (VDR's target gene) and autophagy-related genes were dose dependent inhibited along with STAT6 interference (Figure 4(b)). Oppositely, induction of STAT6 by IL4 upregulated those genes (Figures 4(c) and 4(e)). Furthermore, silencing VDR expression interfered the inductive effect of STAT6 on those autophagy-related genes (Figure 4(d)). These results showed that STAT6 positively regulated autophagy based on VDR.

### 3.4. STAT6 Promotes the Nuclear Translocation of VDR to Activate Its Targets' Transcription

**Figure 5:**
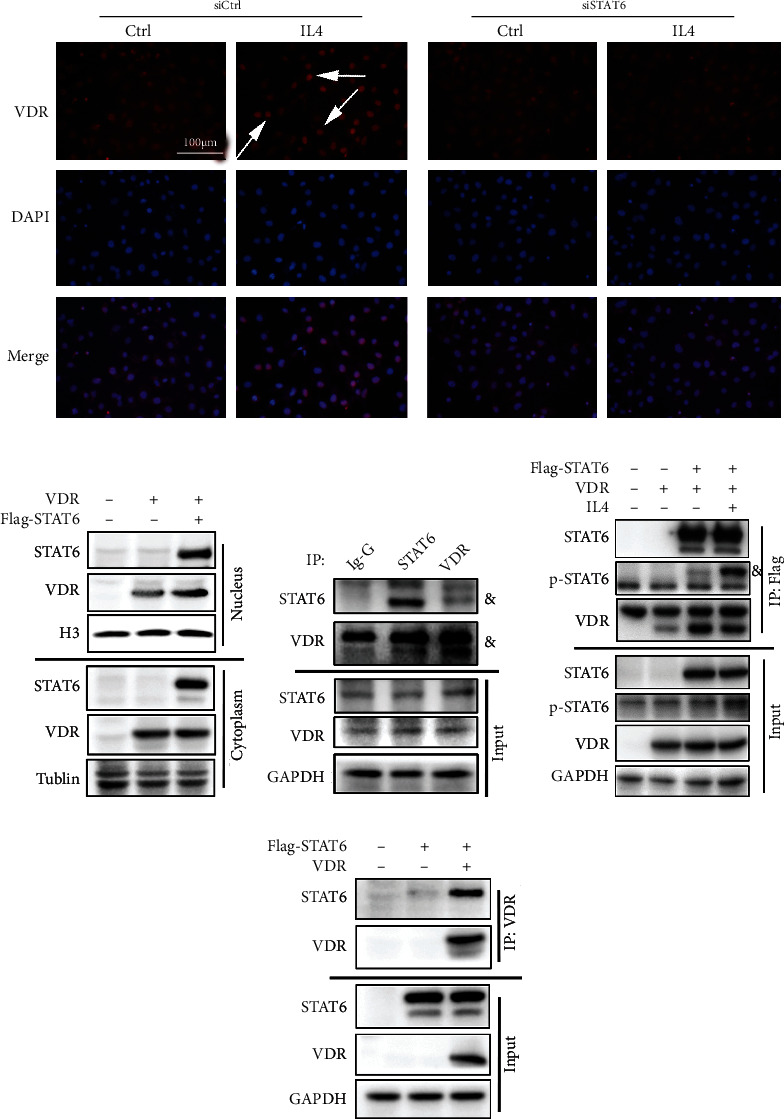
The interaction of STAT6 and VDR. (a) Immunofluorescence staining of VDR in the cells with or without siSTAT6 20 nM 48 h transfection and IL4 (20 ng/ml, 24 h) treatment. Nuclei were stained with DAPI (blue). (b) The protein expression of STAT6 and VDR in the nucleus and cytoplasm after transfection of VDR or STAT6 plasmid in BEAS-2B cells. (c) The protein complex of STAT6-VDR in BEAS-2B cells was assayed by immunoprecipitation to analyze the association between STAT6 and VDR at the endogenous level (and indicate the target bind). (d) HEK293T cells overexpressed different combinations of Flag-STAT6 and VDR as indicated. Exogenous STAT6 interacted with VDR was assayed by immunoprecipitation with Flag beads. (e) HEK293T cells were overexpressed with indicated plasmids (Flag-STAT6 and VDR), and the interaction between VDR and STAT6 was detected by immunoprecipitation using agarose beads along with VDR antibodies.

As well known to us, VDR is a classical nuclear receptor, which transcripts downstream genes after translocation to nucleus. Thus, the location of VDR was detected to explain why there were no observed changes in VDR itself along with STAT6 activation. Immunofluorescence staining was first performed to identify the location of VDR with or without STAT6 activation. The results showed that after treating with IL4, VDR was more detected in nucleus (Figure 5(a)). To further confirm this effect, BEAS-2B transfected with plasmids of VDR or Flag-STAT6 were subjected to separate nuclear and cytoplasmic protein. Both nuclear and cytoplasmic protein lysates were subjected to immunoanalysis. The result showed that VDR had higher expression in nucleus with STAT6 cotransfection rather than VDR transfection only, which suggesting STAT6 activation promoted VDR nuclear translocation (Figure 5(b)). Next, the immunoprecipitation (IP) study was performed to identify the interaction between STAT6 and VDR. The results showed that there is an immune interaction between STAT6 and VDR, as proved both in endogenous and exdogenous levels (Figures 5(c)–5(e)). All these data showed that STAT6 and VDR could bind with each other; thus, STAT6 activation could positively regulate VDR signals through promoting its nuclear translocation.

### 3.5. Silencing VDR Abolishes the Inductive Effects of STAT6 on Nrf2 Signaling Pathway

**Figure 6:**
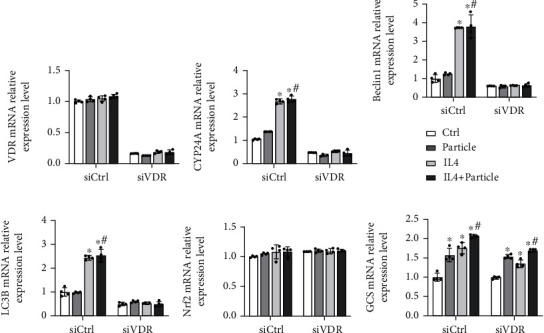
Autophagy-related genes and Nrf2 signaling pathway were decreased with blockade of VDR. BEAS-2B cells were transfected with either Ctrl siRNA or VDR siRNA for 24 h in serum-free medium and then received indicated medium from THP-1 cells (Ctrl and particle groups) with or without IL4 treatment for another 24 h. The total RNA was extracted, and qRT-PCR assay was employed to measure the mRNA expression of (a) VDR, (b) CYP24A, (c) Beclin1, (d) LC3B, (e) Nrf2, and (f) GCS. Results were expressed as means ± SD (*n* = 4; ^∗^*P* < 0.05, Ctrl vs. treatment groups; ^#^*P* < 0.05, P vs. IL4+P).

After unveiling the interaction between STAT6 and VDR, the effect of VDR on particle-induced injury was assessed *in vitro*, using a siRNA-based knockdown approach. THP-1 cells treated with or without particles were cultured for 24 hours. Then, the medium was harvested to treat BEAS-2B cells. BEAS-2B cells with indicated treatment were subjected to qRT-PCR analysis. As shown in Figure 6, activating STAT6 by IL4 upregulated VDR target gene, autophagy-related genes (Beclin1 and LC3B), and Nrf2 signaling pathway as expected. However, these effects were impaired with VDR siRNA transfection. All these data indicated that STAT6 could regulate Nrf2 signaling based on VDR.

### 3.6. STAT6 Protects against Oxidative Damage Based on VDR

**Figure 7:**
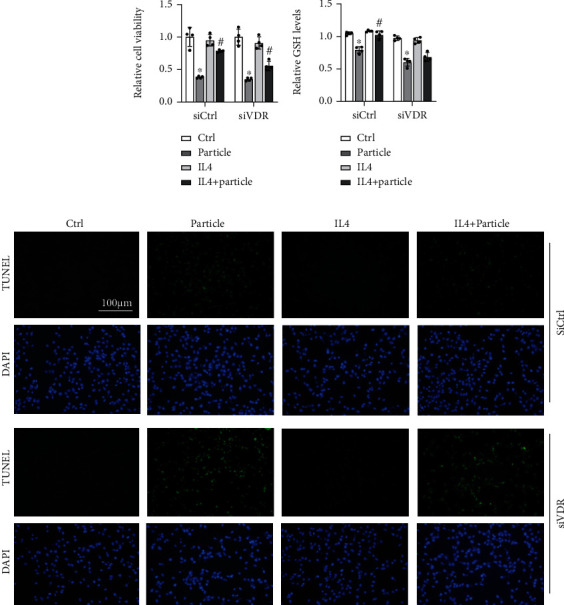
STAT6 protects against oxidative damage through VDR. BEAS-2B cells were transfected with either Ctrl siRNA or VDR siRNA for 24 h in serum-free medium followed by particle medium exposure with or without IL4 24 h administration. (a) The viability of BEAS-2B cells was measured, and results were shown as means ± SD (*n* = 4; ^∗^*P* < 0.05, Ctrl vs. particle; ^#^*P* < 0.05, particle vs. IL4+particle). (b) The GSH levels in the indicated group were detected. Results are expressed as means ± SD (*n* = 4; ^∗^*P* < 0.05, Ctrl vs. P; ^#^*P* < 0.05, P vs. IL4+P). (c) Representative photographs of TUNEL assay staining in BEAS-2B cells from the indicated group were shown.

Prior experiments have identified the importance of STAT6 to particle-induced lung oxidative damage and uncovered its regulation on VDR-autophagy-Nrf2 signaling pathway. Next, the protection of STAT6 against oxidative damage via VDR was further confirmed. MTT assay was used to evaluate the loss of viable cell. Specifically, particle medium treatment caused approximately 50% decrease of epithelial cells' viability, while it can be attenuated by activating STAT6. Knockdown VDR diminished this prevention (Figure 7(a)). Consistently, the decreased GSH levels caused by particle medium were restored by STAT6 activation. However, blocking VDR expression dismissed this effect (Figure 7(b)). Next, the cell apoptosis was performed as well. Particle medium treatment dramatically increased the cell apoptosis, which improved by IL4 treatment, while siVDR weakens the ability of STAT6 antiapoptosis capability as expected (Figure 7(c)). Overall, these data indicated that loss of VDR diminished the oxidation resistance of STAT6.

## 4. Discussion

**Figure 8:**
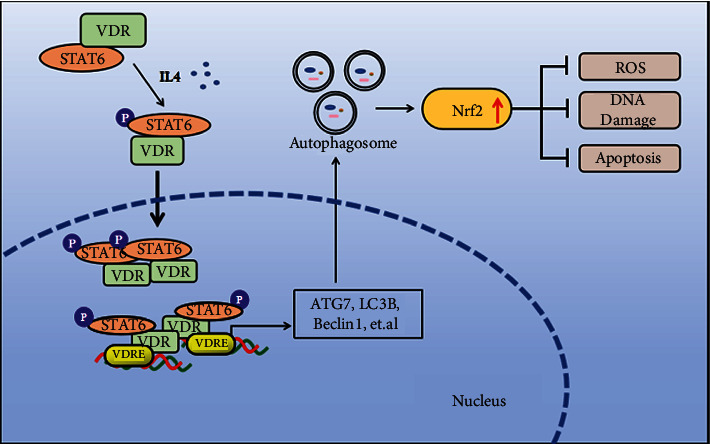
Proposed model for the STAT6/VDR axis mitigates lung inflammatory. STAT6 activation enhances the translocation of VDR to the nucleus, which increased the transcription of autophagy-related genes and then induced Nrf2 signaling, preventing oxidative stress, DNA damage, and apoptosis.

STAT6 has been well studied in most immune responses, but the concept of STAT6-directed protection against airway inflammatory injury has remained largely unexplored. In this study, we elucidated that modulation of STAT6 is essential to combat the damage of oxidative stress and inflammation. Specifically, we found that genetic ablation of STAT6 worsens particle-induced lung injury *in vivo* mainly by impairing the capacity of oxidative resistance of the lungs. Remarkably, we found that STAT6 positively regulates Nrf2 by inducing autophagy. We also observed that STAT6 mechanically promotes VDR nuclear translocation, which transcriptionally activates autophagy-related genes, such as ATG7, Beclin1, and LC3B, further inducing the process of autophagy (Figure 8).

Vitamin D receptor (VDR) is widely distributed in many kinds of cells, and it participates in regulating multiple pathophysiologic processes, such as anti-inflammation activities, infection prevention, and cancer prevention [31–34]. Previous studies have reported that VDR acts to minimize tissue inflammation by mitigating cell death, which prevents the release of intracellular inflammatory mediators and maintains tissue barrier integrity [35, 36]. VDR deficiency results in impaired autophagy and enhanced cell death, particularly in cardiomyopathy, type 2 diabetes, ischemia-reperfusion injury, microbial infections, and cancers [37, 38]. By contrast, VDR activation downregulates the NF-*κ*B signaling pathway, which inhibits COX-2 expression and attenuates inflammation [39]. Furthermore, our previous studies have shown that induction of VDR by its agonist, namely, calcitriol, can activate the Nrf2 signaling pathway through noncanonical mechanisms of autophagy, which attenuate particle-induced lung tissue oxidative stress and cell apoptosis [40]. Mechanically, VDR is reportedly a master transcriptional regulator of autophagy both in the normal mammary gland and BC cells [41].

In this study, we demonstrated that STAT6 activation attenuates particle-induced lung oxidative damage through the Nrf2 signaling pathway. On the basis of established evidence that Nrf2 can be noncanonically activated by autophagy, we further found that STAT6 positively regulates autophagy. We clarified that STAT6 activates autophagy through VDR and also elucidated the regulation of STAT6 in VDR. In detail, STAT6 activation assists in VDR translocation to the nucleus, and then, it transcripts target genes that induce autophagy-related components, such as ATG7 and Beclin1. Thus, we hypothesized that the antioxidative and anti-inflammatory effects of STAT6 may be through VDR. To verify this hypothesis, we employed VDR siRNA. As expected, when VDR expression was blocked, the Nrf2 signaling pathway was suppressed along with the downregulation of autophagy, and the cell oxidative damage caused by particle exposure was amplified.

Overall, our data revealed the role and mechanism of STAT6 in particle-induced pulmonary oxidative damage. Although STAT6 is a crucial factor in immunoregulation, most studies have focused on its function in autoimmune response, and its role in preventing oxidative stress has been poorly explored. Our *in vivo* studies provided definitive evidence that modulation of STAT6 is correlated with particle-induced pulmonary oxidative injury. Further, we unveiled the potential underlying mechanism of this effect, and this information may provide a theoretical basis and a clinical target for the treatment of lung injury.

## 5. Conclusion

Our study demonstrated that STAT6 is a vital factor against inflammatory injury. STAT6 activation enhances the translocation of VDR to the nucleus, a process that increases the transcription of autophagy-related genes and then induces Nrf2 signaling, thereby preventing oxidative stress, DNA damage, and apoptosis.

## Figures and Tables

**Table 1:** The primer sequences used in this study.

Primers sequence (5′3′)		
m-Nrf2	Forward	CTCAGCATGATGGACTTGGA
	Reverse	TCTTGCCTCCAAAGGATGTC

m-GCS	Forward	TCCCATGCAGTGGAGAAGAT
	Reverse	AGCTGTGCAACTCCAAGGAC

m-NQO1	Forward	GGTAGCGGCTCCATGTACTC
	Reverse	AGACCTGGAAGCCACAGAAA

m-STAT6	Forward	CTCTGTGGGGCCTAATTTCCA
	Reverse	CATCTGAACCGACCAGGAACT

m-Arg-1	Forward	CGCCTTTCTCAAAAGGACAG
	Reverse	TTTTTCCAGCAGACCAGCTT

m-VDR	Forward	CTGTGGCAGCCAAGACTACA
	Reverse	GCAGCACATGTTCTTCCTCA

m-CYP24A	Forward	CAAGGCAACAGTTCTGGGTGA
	Reverse	GTCTTCACTGGATCCCAACAC

m-ATG7	Forward	AGGCACCCAAAGACATCAAG
	Reverse	GCACTGAACTCCAACGTCAA

m-Beclin1	Forward	GAGCCATTTATTGAAACTCGCCA
	Reverse	CCTCCCCGATCAGAGTGAA

m-LC3B	Forward	CCGCACCTTCGAACAAAGAG
	Reverse	AAGCTGCTTCTCACCCTTGT

m-*β*-actin	Forward	AAGGCCAACCGTGAAAAGAT
	Reverse	GTGGTACGACCAGAGGCATAC

h-VDR	Forward	CCAGTTCGTGTGAATGATGG
	Reverse	AGATTGGAGAAGCTGGACGA

h-CYP24A	Forward	TCTCAAGAAACAGCACGACACCC
	Reverse	GCACCGACTCAAAGGAACCCAAC

h-Beclin1	Forward	TGGACACGAGTTTCAAGATCC
	Reverse	AAATGGCTCCTCTCCTGAGTT

h-LC3B	Forward	CCGCACCTTCGAACAAAGAG
	Reverse	AAGCTGCTTCTCACCCTTGT

h-Nrf2	Forward	ACACGGTCCACAGCTCATC
	Reverse	TGTCAATCAAATCCATGTCCTG

h-GCS	Forward	GACAAAACACAGTTGGAACAGC
	Reverse	CAGTCAAATCTGGTGGCATC

h-GAPDH	Forward	CTGACTTCAACAGCGACACC
	Reverse	TGCTGTAGCCAAATTCGTTGT

## Data Availability

All data used to support the findings of this study are available from the corresponding author upon request.

## Abbreviations

MDA: Malondialdehyde
GSH: Glutathione
VDR: Vitamin D receptor
Nrf2: Nuclear factor (erythroid-derived 2)-like 2
ROS: Reactive oxygen species.
BALF: Bronchial alveolar lavage fluid.
